# Chromium Exposure and Hygienic Behaviors in Printing Workers in Southern Thailand

**DOI:** 10.1155/2015/607435

**Published:** 2015-09-13

**Authors:** Somsiri Decharat

**Affiliations:** Department of Industrial Hygiene and Health Science, Faculty of Health and Sports Science, Thaksin University, Phatthalung 93210, Thailand

## Abstract

*Objectives.* The main objective of this study was to assess the chromium exposure levels in printing workers. The study evaluated the airborne, serum, and urinary chromium levels and determines any correlation between level of chromium in specimen and airborne chromium levels.* Material and Methods.* A cross-sectional study was conducted with 75 exposed and 75 matched nonexposed subjects. Air breathing zone was measured by furnace atomic absorption spectrophotometer. Serum and urine samples were collected to determine chromium levels by graphite furnaces atomic absorption spectrometer chromium analyzer.* Results and Discussion.* The printing workers' urinary chromium levels (6.86 ± 1.93 *μ*g/g creatinine) and serum chromium levels (1.24 ± 1.13 *μ*g/L) were significantly higher than the control group (*p* < 0.001 and *p* < 0.001). Work position, duration of work, personal protective equipment (PPE), and personal hygiene were significantly associated with urinary chromium level and serum chromium levels (*p* < 0.001 and *p* < 0.001). This study found a correlation between airborne chromium levels and urinary chromium levels (*r* = 0.247, *p* = 0.032). A multiple regression model was constructed. Significant predictors of urinary and serum chromium levels were shown in this study.* Conclusion.* Improvements in working conditions, occupational health training, and PPE use are recommended to reduce chromium exposure.

## 1. Introduction

Chromium exits in two stable oxidation states, namely, the trivalent (III) and hexavalent (VI) forms. Hexavalent Cr is more toxic than trivalent Cr and the toxicological impact is due to its oxidizing ability and high solubility [1–4]. Chromium (VI) is capable of damaging the skin due to its high penetration power and ability to form free radicals. Printing workers operated by five separate and distinct processes, lithography, letterpress, flexography, gravure, and screen printing [5, 6]. The International Agency for Research on Cancer (IARC) classified occupational exposure in the printing industry as possibly carcinogenic to humans [7]. The toxicity of chromium within the cell may result from damage to cellular components during the hexavalent to trivalent chromium reduction process, by generation of free radicals, including DNA damage [8, 9]. Recent studies indicate a biological relevance of nonoxidative mechanisms in Cr(VI) carcinogenesis [10]. Printing workers are potentially exposed to hexavalent Cr when involved in the production use of chromate pigments, chromate paints, and printing inks. Studies of workers in the chromium pigment, chrome plating, and ferrochromium industries showed a statistically significant association between worker exposure to Cr(VI) and lung cancer and nasal and sinus cancer [8–12]. More recent studies also disclosed excess risk of lung cancer death resulting from occupational exposure to Cr(VI) compounds [13, 14]. Direct contact of Cr(VI) compounds with intact skin can induce chromium dermatitis or sensitization. In the case of preexisting small skin lesions, impact of hexavalent chromium compounds will lead to slowly healing chromium ulcers. The objectives of this study were to determine and evaluate the level of urinary chromium and level of serum chromium among printing workers exposed to chromium from printing factories and compare them with the nonexposed group and to describe worker behaviors and evaluate them in terms of their possible role in worker contamination and transfer of chromium to the body and to determine any correlation between level of urinary chromium and level of serum chromium and airborne chromium levels.

## 2. Material and Methods

### 2.1. Study Site and Study Subjects

Data for this cross-sectional study were collected by sampling from 75 printing workers (49 males; 26 females) in southern Thailand from January to September 2014. Seventy-five printing workers were recruited from 16 printing factories. In this study, the process of printing production included prepress process (digitization, preflight, imposition, digital proof ting, process film making, plating making, and plate proofing), press process (print preparation and printing) and postpress process (surface decoration, coating, hot stamping, embossing, forming, book making packing). Inclusion criteria for the study subjects were as follows: printing work, aged 20–54 years, in direct contact with heavy metals, and working in printing factories for at least one year prior to the study; persons who had been in occupational contact with chromium dust during printing production; persons who agreed to participate in the study and who signed the informed consent form. The nonexposed group (75 persons) matched to exposed subjects by age and sex were recruited from the printing workers who worked at the same printing factories but had not had occupational contact chromium (35 office workers, 25 drivers, and 15 cleaners, resp.).

### 2.2. Blood Serum Collections

The collected venous blood samples were placed into sterile, closed tubes. After two hours, stay blood samples were centrifuged at 3500 rpm for 10 min and separated sera were put in closed plastic laboratory vessels and kept at −18°C in the fridge. Blood serum samples, obtained from subjects of participation, were used to determine the chromium levels by the analytical procedure.

### 2.3. Urine Collections

The 150 subjects (75 exposed and 75 unexposed) were interviewed using structured questionnaire interviews. Spot urine samples (30 mL) were collected, which extends from the time the subjects goes to bed through the first urination of the morning. The urine samples were kept in polypropylene sampling vessels and stored at −20°C prior to analysis.

### 2.4. Questionnaire

In the questionnaire interviews, detailed descriptive information was collected, including personal descriptive characteristics, occupational life styles, working positions, working environment, and personal hygiene. Direct observations were also made and recorded to confirm the questionnaire interviews. At the end of shifts, the subjects were also interviewed.

### 2.5. Airborne Chromium Collections

Personal samples were taken from 75 printing workers in 16 printing factories during January–September 2014. Regarding the instrument for air sampling, personal pumps (Model 224-PCXR8; SKC Inc., Eighty Four, PA, USA) were calibrated at 2 L/min before and after sampling, containing a mixed cellulose ester membrane filter (pore size: 0.8). Sampling was carried out for the regular work duration of 8 h. The air sampling equipment was fitted to the subject at the start of the day, removed, or switched off during the break and finally removed at the end of the day. The procedure was specified by the NIOSH method 7024/1994 [15]. Air samples were collected at the breathing zone and at the printing workplace. Airborne lead concentration was measured by furnace atomic absorption spectrophotometer (FAAS).

## 3. Laboratory Analysis

### 3.1. Determination of Chromium in Air Samples

Air sample filters and field blanks were subjected to slow wet acid digestion in accordance with the NIOSH standard analytical method 7024 [15]. Each sample solution was diluted with 0.1 M nitric acid to 10 mL in a volumetric flask prior to chemical analysis. The concentrations of Cr in the digested breathing zone air were determined using FAAS following the NIOSH method 7024 [15]. Working standards for Cr were diluted appropriately in 0.01 M nitric acid (1% (v/v) as a stabilizer). Aqueous standard solutions with concentrations of Cr were run, which gave the required standard calibration curves. The concentrations of Cr in the digested breathing zone air samples were assayed in triplicates using FAAS at optimized operational conditions. This was subsequently obtained directly from the standard calibration graphs after correction of the absorbance for the signals from appropriate reagent blanks. Airborne levels of Cr were expressed as micrograms (*μ*g) per cubic meter (m^3^) of air over an 8-hour time-weighted average (TWA).

### 3.2. Quantification of Chromium in Serum

The serum sample is added by Mg(NO_3_)_3_. The sample was dried by lyophilization and then ashed and dissolved in 0.1 HCl. This method of serum chromium determination was modified from that of Randall and Gibson, 1987 [16].

### 3.3. Quantification of Chromium in Urine

Total chromium (Cr) standards were prepared from the commercial stock solution of 1000 *μ*g/mL in 0.01 M nitric acid by successive dilution with distilled and deionized water. These standard solutions were subsequently used to generate the appropriate standard calibration curves. Similarly for Cr analysis, aliquots of 10 *μ*L of the diluted urine samples were introduced directly into a pyrolytically coated graphite furnace tube and, with an equal volume of 10 *μ*L matrix modifier mixture (0.6% palladium nitrate and 0.15% *m*/*v* magnesium nitrate in 0.01 M nitric acid), were automatically injected sequentially [17]. The concentrations of Cr were obtained directly from the calibration graphs after automatic correction of the absorbance of the signal from appropriate reagent blanks. Creatinine levels were analyzed in all spot urine samples using the alkaline picrate method, which was based on a modified Jaffe reaction [18].

### 3.4. Validation of Chromium in Air, Serum, and Urine Analyses

All samples were analyzed using adequate quality control procedure to ascertain reliability of the results. The reagents that were used through the analytical procedure were of high purity analytical grade. The main instrument parameter for graphite flame atomic absorption spectrometry (GFASS) and flame atomic absorption spectrometry (FASS) was optimized separately for each metal.

The determinations of chromium in the serum were diluted five times with deionized water. A stock stand containing 1000 mg·L^−1^ of chromium was obtained from Sigma chemical Co. (St. Louis, MO).

Quality control was further ascertained by inter laboratory comparison of the levels of chromium in five sets of representative breathing zone air, serum, and urinary samples. The rage of linearity was also determinate checking the linear regression coefficient (*R* ^2^) of calibration values. It was considered acceptable when *R* ^2^ was 0.995. The validity of the method was further assured by method cross check and replication analysis. The calculated precision was within 8% relative standard deviation (RSD).

### 3.5. Ethical Approval

This study was approved by the ethical committee of Thaksin University Review Board. All of the participants received a clear explanation of the purpose of this study and agreed to participate using signed consent forms.

### 3.6. Statistical Analysis

Descriptive statistics were used to present the airborne, serum, and urine concentration results. The independent *t*-test was used to compare the means of continuous variables. Pearson's test was used to test the association of airborne and specimen levels. Normally distributed data were compared using the Student's *t*-test. Statistical significance was defined as *p* < 0.05. The regression analysis was used for estimating the relationships among variables in this study.

## 4. Results

### 4.1. Distribution of Demographic Characteristic of Printing Workers

One hundred fifty subjects participated in the present study. Most of the exposed subjects (65.3%) were male and 53.3% were aged between 20–34 years. All subjects were Buddhists. Most of the exposed subjects had bachelor's degree (41.3%), similar to the control subjects (36.4%). More control subjects smoked cigarettes and drank alcoholic beverage compared to exposed subjects.

### 4.2. Airborne Chromium Level, Serum Chromium Levels, and Urinary Chromium Levels

The mean airborne chromium level was 7.20 ± 2.86 *μ*g/m^3^ (range: 1–12 *μ*g/m^3^) where 82.67% exceeded the standard of The Occupational Safety and Health Administration (OSHA) [19] level of 5 *μ*g/m^3^ as Cr(VI) *µ*g/m^3^ and PEL for chromic acid and chromates and (8-hour TWA) as air workplace. The mean serum chromium levels of the exposed and control subjects were significantly different at *p* value of <0.001, while the urinary chromium levels of the exposed and control subjects were significantly different at *p* value of <0.001 (Table 1).

### 4.3. Correlation between Level of Urinary Chromium and Level of Serum Chromium and Airborne Chromium Levels

**Figure 1:**
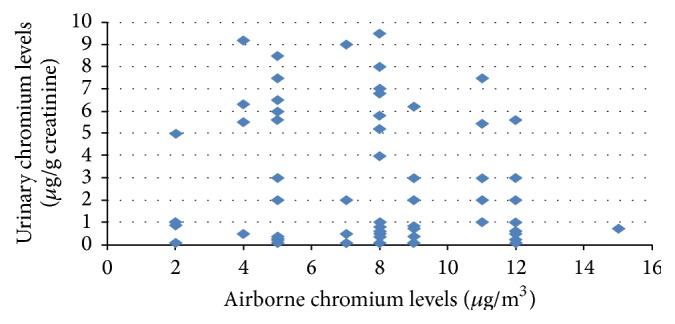
The correlation plot of airborne chromium levels (personal sampling) versus printing workers' urinary chromium levels.

**Figure 2:**
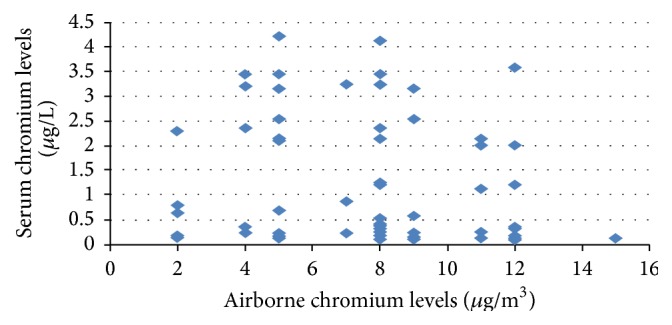
The correlation plot of airborne chromium levels (personal sampling) versus printing workers' serum chromium levels.

There were significant correlations between the sampling breathing zone at the chromium storage subjects and levels of chromium in urine (*r* = 0.247, *p* < 0.032) (Figure 1) and levels of chromium in serum (*r* = 0.166, *p* = 0.158) (Figure 2).

### 4.4. Occupational Lifestyles, PPE, Personal Hygiene of Workers, and Chromium Levels

Male workers had significantly higher serum chromium levels and urinary chromium levels than those female printing workers, at *p* values of <0.001 and 0.039, respectively. Smoking workers had significantly higher urinary chromium levels than those nonsmoke printing workers, at *p* value of 0.034. It was found that duration of work, hours worked per day, days worked per week, position, use of personal protective equipment (PPE), eating snacks or drinking water during work, washing hands before lunch, and washing hands after work were significantly different, at *p* < 0.05 (Table 2). Printing workers who had worked > 5 years had significantly higher serum chromium levels and urinary chromium levels than those who had worked ≤ 5 years (*p* < 0.001). Printing workers who had worked > 8 hours per day and >6 days per week had significantly higher serum chromium levels and urinary chromium levels than those who had worked ≤8 hours per day and ≤6 days per week (*p* = 0.012 and *p* < 0.001, resp.). The results indicated that the mean serum chromium levels and urinary chromium levels among work positions were significantly different (*p* < 0.001). Printing workers who used mask and/or gloves had significantly lower serum chromium levels and urinary chromium levels than those who did not. Workers who always ate snacks had significantly higher serum chromium levels and urinary chromium levels than those who sometimes ate them. Printing workers who always washed their hands after work had significantly lower serum chromium levels and urinary chromium levels than those who sometimes did so.

To predict the serum chromium levels of printing workers, a regression model was constructed, as in the first equation. There were significant influences between the independent variables (smoking, hours per day, years of work, positions, and using mask) and the serum chromium levels; the entire *R* ^2^ was 0.711, indicating that the urinary chromium levels could be interpreted into 71.1% of the independent variables. The independent variables (using mask) affected the mediator (serum chromium levels) (*p* < 0.001). In the second equation, there were significant influences between the independent variables (smoking, hours per day, years of work, positions, and using gloves) and the serum chromium levels; the entire *R* ^2^ was 0.418, indicating that the urinary chromium levels could be interpreted into 41.8% of the independent variables. The independent variables (hours per day and using gloves) affected the mediator (serum chromium levels) (*p* < 0.001 and *p* = 0.024, resp.). In the third equation, there were significant influences between the independent variables (smoking, hours per day, years of work, positions, and eating snacks or drinking water at work) and the serum chromium levels; the entire *R* ^2^ was 0.386, indicating that the urinary chromium levels could be interpreted into 38.6% of the independent variables. The independent variables (hours per day) affected the mediator (serum chromium levels) (*p* < 0.001).

In the fourth equation, there were significant influences between the independent variables (smoking, hours per day, years of work, positions, and washing hands before lunch) and the serum chromium levels; the entire *R* ^2^ was 0.425, indicating that the urinary chromium levels could be interpreted into 42.5% of the independent variables. The independent variables (hours per day, positions, and washing hands before lunch) affected the mediator (serum chromium levels) (*p* < 0.001, *p* = 0.049, and *p* = 0.015, resp.). In the fifth equation, there were significant influences between the independent variables (smoking, hours per day, years of work, positions, and washing hands after work) and the serum chromium levels; the entire *R* ^2^ was 0.377, indicating that the urinary chromium levels could be interpreted into 37.7% of the independent variables. The independent variables (hours per day) affected the mediator (serum chromium levels) (*p* < 0.001) (Table 3).

To predict the urinary chromium levels of printing workers, a regression model was constructed, as in the first equation. There were significant influences between the independent variables (smoking, hours per day, years of work, positions, and using mask) and the urinary chromium levels; the entire *R* ^2^ was 0.542, indicating that the urinary chromium levels could be interpreted into 54.2% of the independent variables. The independent variables (hours per day, years of work, and positions) affected the mediator (urinary chromium levels) (*p* = 0.006, *p* = 0.001 and *p* = 0.011, resp.). In the second equation, there were significant influences between the independent variables (smoking, hours per day, years of work, positions, and using gloves) and the urinary chromium levels; the entire *R* ^2^ was 0.508, indicating that the urinary chromium levels could be interpreted into 50.8% of the independent variables. The independent variables (hours per day, years of work, and positions) affected the mediator (urinary chromium levels) (*p* < 0.001, *p* < 0.001, and *p* = 0.036, resp.).

In the third equation, there were significant influences between the independent variables (smoking, hours per day, years of work, positions, and eating snacks or drinking water at work) and the urinary chromium levels; the entire *R* ^2^ was 0.532, indicating that the urinary chromium levels could be interpreted into 53.2% of the independent variables. The independent variables (hours per day, years of work, and eating snacks or drinking water at work) affected the mediator (urinary chromium levels) (*p* < 0.001, *p* < 0.001, and *p* = 0.017, resp.). In the fourth equation, there were significant influences between the independent variables (smoking, hours per day, years of work, positions, and washing hands before lunch) and the urinary chromium levels; the entire *R* ^2^ was 0.532, indicating that the urinary chromium levels could be interpreted into 53.2% of the independent variables. The independent variables (hours per day, years of work, positions, and washing hands before lunch) affected the mediator (urinary chromium levels) (*p* = 0.001, *p* < 0.001, *p* = 0.005, and *p* = 0.030, resp.). In the fifth equation, there were significant influences between the independent variables (smoking, hours per day, years of work, positions, and washing hands after work) and the urinary chromium levels; the entire *R* ^2^ was 0.492, indicating that the urinary chromium levels could be interpreted into 49.2% of the independent variables. The independent variables (hours per day, years of work, and positions) affected the mediator (urinary chromium levels) (*p* < 0.001, *p* = 0.001, and *p* = 0.010, resp.) (Table 4).

## 5. Discussion

The mean factory airborne chromium level was 7.20 ± 2.86 *μ*g/m^3^ (range 1–12 *μ*g/m^3^). Sixty-two of 75 exposed subjects (82.67%) had levels that exceeded the accepted 8-hour time-weighted average (TWA) exposure limit of 5 micrograms of Cr(VI) per cubic meter of air (5 *μ*g/m³) recommended by Occupational Safety and Health Administration (OSHA) [19]. However, an analysis of lung cancer risk suggests a potential excess risk of death from lung cancer among US workers exposed to the previous permissible exposure limit (PEL) for Cr(VI) of 52 *μ*g/m³ [20].

The concentration of an element in serum reflects the accumulation of this element in the organism [21], while the urinary excretion of chromium has already been considered as a possible biological marker in occupational exposure to hexavalent chromium [22–25].

The results of the present study showed that the serum chromium levels and urinary chromium levels in these printing workers were higher than those in the matched control subjects (*p* < 0.001 and *p* < 0.001, resp.). This is supported by Kornhauser et al. [21], who reported serum chromium levels in high exposed to chromium group were higher as compared to control group and showed increased urine excretion of chromium in high exposed to chromium group.

Among printing workers in the present study, the mean serum chromium levels and urinary chromium levels were 1.24 ± 1.13 *μ*g/L (range: 0.0–4.21 *μ*g/L) and 6.86 ± 1.93 *μ*g/g creatinine (range: 0.1–9.5 *μ*g/g creatinine), respectively. All printing workers had serum chromium levels of <2 *μ*g/100 mL [26] and urinary chromium levels of <30 *μ*g/g creatinine, respectively [27], the ATSDR and ACGIH recommended biological exposure index for serum chromium levels and urinary chromium levels.

This study found a correlation between airborne chromium levels and urinary chromium levels (*r* = 0.247, *p* = 0.032). This result agreed with Faridah et al. [28], who reported a correlation between airborne chromium levels and urinary chromium levels in the tannery workers plant. A strong positive correlation was observed, suggesting that urinary chromium levels were direction influence. The presence of chromium in the specimen of the printing workers may be due to contribution from the breathing zone air through inhalation. It was previously reported that the level of an element in serum reflects the accumulation of this element in the organism [22], but it could not be recommended as a reliable assessment of the health risk related with occupational exposure.

In this study, we found that many factors influence increased serum chromium levels and urinary chromium levels. Workers who worked in printing process had higher serum chromium levels and urinary chromium levels than prepress and postpress process. Although most printing workers worked inside factories, all workers brought them into continuous contact with chromium, which is reflected in the higher serum chromium levels and urinary chromium levels among the printing workers compared to the other position. The result of the present study was similar to that of Kornhauser et al. [21], in which the serum chromium levels and urinary chromium levels of tannery workers differed by job type (dying, drying, and finishing).

With regard to working duration, it was found that mean serum chromium levels and urinary chromium levels differed significantly; printing workers who had worked > 5 years had significantly higher serum and urinary chromium levels than those who had worked ≤ 5 years. In addition, for hours workers per day and days worked per week, mean serum chromium levels and urinary chromium levels differed significantly; printing workers who had worked > 8 hours per day and > 6 days per week had significantly higher serum and urinary chromium levels than those who had worked ≤ 8 hours per day and ≤ 6 days per week (*p* < 0.001 and *p* < 0.001, resp.). This may be due to printing workers' long term exposure to chromium, leading to its accumulation in their bodies, due to the lack of appropriate prevention measures [22].

Printing workers were considered informal workers; some used their work areas during their breaks. Therefore, personal working habits and the conditions at the workplace area seem to affect the exposure and cause difference [29].

The use of PPE at work can help prevent contamination. Printing workers who used masks and gloves had significantly lower serum and urinary chromium levels than those who did not [30]. However, the personal hygienic practices were not appropriate for field work in these printing factories. Cotton masks used by printing workers may accumulate chromium dust on their surface. In addition, fume of chromium and other chemicals (VOC) may penetrate though a cotton mask and gain access to a printing worker's airway. The results have indicated that chromium is a risk factor where there are inadequate engineering controls, industrial hygiene, and work practice, particularly in occupational risk [18].

The present study supported by Chuang et al. [31] and Tawichascri et al. [32] has indicated that poor behavior plated a role in accumulation of airborne chromium among workers. Thus the combinations of poor work practices and safety behavior among workers, the limited training and education on safety procedures, may have immensely contributed to elevated levels of chromium in both the breathing zone air and urinary that were measured in these facilities. The present study was supported by Lumens et al. (1993) [33], who reported that, in addition to individual difference in hygiene behavior, general hygiene condition also has an impact on uptake of chromium. That showed a significant impact of hygiene behavior on the variance in urine chromium levels (*R* ^2^ = 0.94, *p* < 0.001).

From observations of work areas, contamination of hands, cloths, hand tool, and working surface with chemical was clearly evident at each work site [34]. Printing workers who always ate snacks or drank water while working had significantly higher serum and urinary chromium levels than those who only did so sometimes. Printing workers who always washed their hand before lunch had significantly lower serum and urinary chromium levels than those who sometimes did. In addition, printing workers who always washed their hand after worked had significantly lower serum and urinary chromium levels than those who sometimes did.

Smoking has a very important confounder because of tobacco contents or contamination by chromium. However, the factor of smoking cigarettes in this study has not demonstrated increased risk for cancer. In addition, these studies found an absence of risk, except at high exposure levels. Sparse data precluded the control of tobacco smoke as a confounder in analyses of the US cohort [35]. Hexavalent chromium is an established lung carcinogen. Luippold and Birk et al. [36, 37] have reported that examining lung cancer mortality among chromate production workers in the US and in Germany was subsequent to significant process changes and enhanced industrial hygiene controls.

## 6. Conclusions

Cr(VI) compounds may be used as pigments in dyes, paints, inks, and plastics. It also may be used as an anticorrosive agent added to paints, primers, and other surface coatings as a known human carcinogen. Thus, this study demonstrated that urinary chromium levels and serum chromium levels were associated with airborne chromium levels and hygiene behaviors of printing workers. This study showed that improving printing workers hygiene habits can reduce urinary mercury levels. This study recommends conducting education and training about personal hygiene to minimize occupational chromium exposure. In addition, engineering controls are also recommended to reduce chromium or other chemical exposures and to reduce the printing worker's cancer hazard to minimal levels. In addition, the limitation in this study was able to determine total chromium in both breathing zone, serum, and urinary sample and we found it difficult to analyse chromium (VI). However, we recommended that further research should distinguish chromium (III) from chromium (VI) because of greater toxicity of chromium (VI).

## Figures and Tables

**Table 1:** Mean airborne levels, serum chromium levels, and urinary chromium levels.

Parameters	Control subjects (*n* = 75)	Exposed workers (*n* = 75)	*p* value
Mean airborne levels (*μ*g/m^3^) ± SD	—	7.20 ± 2.86	

Mean serum chromium levels (*μ*g/L) ± SD	0.12 ± 0.07 (0.1–0.26)	1.24 ± 1.13 (0.1–4.21)	0.031

Mean urine chromium levels (*μ*g/g creatinine) ± SD	1.06 ± 1.11 (0.1–1.20)	6.86 ± 1.93 (0.1–9.5)	<0.001

Significant at *p* < 0.05.

**Table 2:** Variables related to printing workers' serum chromium levels and urinary chromium levels.

Characteristics	Number of exposed printing workers (*n* = 75)	Mean ± SD of serum chromium levels (*μ*g/L)	*p* value	Mean ± SD of urinary chromium levels (*μ*g/g creatinine)	*p* value
*General characteristics *					
Sex					
Male	49 (65.3)	1.47	0.001^*∗*^	3.36 ± 1.90	0.039^*∗*^
Female	26 (34.7)	0.59 ± 0.19		1.91 ± 0.70	
Age (years)					
20–30	40 (53.3)	0.67 ± 0.52	0.067	1.71 ± 0.05	0.425
>30–40	24 (32.0)	1.47 ± 1.29		2.90 ± 1.80	
>40–50	11 (14.7)	1.62 ± 1.21		1.92 ± 1.62	
Education level					
Secondary school	15 (20.0)	1.29 ± 1.13	0.614	0.31 ± 0.30	0.214
Vocational school	29 (38.7)	0.96 ± 1.17		0.36 ± 0.21	
Bachelor's degree or higher	31 (41.3)	1.19 ± 1.20		0.41 ± 0.30	
Income (baht)					
≤9000	48 (64.0)	1.16 ± 1.02	0.780	2.83 ± 1.19	0.926
>9000	27 (36.0)	1.28 ± 1.01		2.90 ± 1.97	
Smoking cigarettes					
Yes	35 (46.7)	1.37 ± 1.31	0.123	3.62 ± 2.17	0.034^*∗*^
No	40 (53.3)	0.92 ± 1.14		2.19 ± 1.55	
Consuming alcohol					
Yes	43 (57.3)	1.07 ± 1.23	0.635	3.05 ± 2.09	0.517
No	32 (42.7)	1.21 ± 1.20		2.61 ± 2.11	

*Occupational lifestyles*					
Duration of work (years)					
>5	21 (28.0)	0.67 ± 0.18	0.012^*∗*^	0.57 ± 0.06	<0.001^*∗*^
≤5	54 (72.0)	1.31 ± 1.20		3.75 ± 0.99	
Hours worked per day					
>8	48 (64.0)	0.56 ± 0.27	<0.001^*∗*^	1.57 ± 0.21	<0.001^*∗*^
≤8	27 (36.0)	2.14 ± 1.30		5.15 ± 2.15	
Days worked per week					
>6	59 (78.8)	0.77 ± 0.02	<0.001^*∗*^	2.11 ± 0.71	<0.001^*∗*^
≤6	16 (21.3)	2.85 ± 0.91		5.59 ± 1.95	
Position					
Press or printing process	58 (77.3)	1.80 ± 1.36	<0.001^*∗*^	4.51 ± 1.30	<0.001^*∗*^
Other positions (prepress and postpress process)	17 (22.7)	0.26 ± 1.60		2.41 ± 0.02	

*Personal protective equipment (PPE)*					
Mask					
Yes	21 (28.0)	2.80 ± 0.842	<0.001^*∗*^	5.37 ± 2.33	<0.001^*∗*^
No	54 (72.0)	0.48 ± 0.582		1.85 ± 2.50	
Gloves					
Yes	45 (60.0)	1.65 ± 1.336	<0.001^*∗*^	3.87 ± 2.9	<0.001^*∗*^
No	30 (40.0)	0.35 ± 0.393		1.34 ± 1.12	

*Personal hygiene of workers*					
Eating snacks or drinking water at work					
Always	57 (76.0)	1.41 ± 1.303	<0.001^*∗*^	3.47 ± 1.95	0.001^*∗*^
Sometimes	18 (24.0)	0.27 ± 0.178		1.92 ± 1.82	
Washing hands before lunch					
Always	35 (46.7)	1.60 ± 1.303	0.002^*∗*^	4.29 ± 3.91	<0.001^*∗*^
Sometimes	40 (53.3)	0.72 ± 1.029		1.60 ± 1.17	
Washing hands after work					
Always	15 (20.0)	2.33 ± 1.308	0.002^*∗*^	5.41 ± 2.43	0.004^*∗*^
Sometimes	60 (80.0)	0.83 ± 1.028		2.21 ± 1.41	

^*∗*^Significant at *p* < 0.05.

**Table 3:** Regression analysis of occupational life style, used PPE, and personal hygiene behavior on serum chromium levels (*n* = 75).

Dependent variable-independent variable	Adjusted *R* ^2^	Standardized beta coefficient	*t* value	Significance
First equation				
Smoking	0.711	0.047	0.718	0.475
Hours per day		0.045	0.505	0.615
Years of work		0.000	−0.008	0.994
Positions		0.111	1.732	0.088
Mask		−0.808	−8.980	0.000^*∗*^

Second equation				
Smoking	0.418	−0.010	−0.114	0.910
Hours per day		0.455	4.117	0.000^*∗*^
Years of work		0.080	0.846	0.400
Positions		0.105	1.092	0.279
Gloves		−0.253	−2.311	0.024^*∗*^

Third equation				
Smoking	0.386	0.005	0.056	0.955
Hours per day		0.554	5.476	0.000^*∗*^
Years of work		0.046	0.475	0.636
Positions		0.122	1.175	0.244
Eating snacks or drinking water at work		−0.130	−1.195	0.236

Fourth equation				
Smoking	0.425	0.117	1.114	0.269
Hours per day		0.444	4.042	0.000^*∗*^
Years of work		0.082	0.865	0.390
Positions		0.180	2.001	0.049^*∗*^
Washing hands before lunch		−0.304	−2.508	0.015^*∗*^

Fifth equation				
Smoking	0.377	−0.011	−0.112	0.911
Hours per day		0.592	5.962	0.000^*∗*^
Years of work		0.043	0.441	0.661
Positions		0.194	1.993	0.050
Washing hands after work		0.062	0.646	0.521

^*∗*^Significant at *p* < 0.05.

**Table 4:** Regression analysis of occupational life style, used PPE, and personal hygiene behavior on urinary chromium levels (*n* = 75).

Dependent variable-independent variable	Adjusted *R* ^2^	Standardized beta coefficient	*t* value	Significance
First equation				
Smoking	0.542	−0.077	−0.906	0.362
Hours per day		0.333	2.833	0.006^*∗*^
Years of work		0.310	3.595	0.001^*∗*^
Positions		0.218	2.599	0.011^*∗*^
Mask		−0.183	−1.559	0.124

Second equation				
Smoking	0.508	−0.089	−1.051	0.297
Hours per day		0.376	3.703	0.000^*∗*^
Years of work		0.343	3.923	0.000^*∗*^
Positions		0.189	2.142	0.036^*∗*^
Gloves		−0.153	−1.519	0.133

Third equation				
Smoking	0.532	−0.058	−0.688	0.493
Hours per day		0.400	4.533	0.000^*∗*^
Years of work		0.328	3.902	0.000^*∗*^
Positions		0.134	1.483	0.143
Eating snacks or drinking water at work		0.553	−2.437	0.017^*∗*^

Fourth equation				
Smoking	0.526	0.014	0.151	0.881
Hours per day		0.342	3.420	0.001^*∗*^
Years of work		0.352	4.104	0.000^*∗*^
Positions		0.235	2.872	0.005^*∗*^
Washing hands before lunch		−0.244	−2.223	0.030^*∗*^

Fifth equation				
Smoking	0.492	−0.090	−1.049	0.298
Hours per day		0.455	5.077	0.000^*∗*^
Years of work		0.319	3.650	0.001^*∗*^
Positions		0.234	2.667	0.010^*∗*^
Washing hands after work		0.006	0.069	0.945

^*∗*^Significant at *p* < 0.05.
